# Cerebral Cortex Expression of *Gli3* Is Required for Normal Development of the Lateral Olfactory Tract

**DOI:** 10.1371/journal.pone.0141525

**Published:** 2015-10-28

**Authors:** Eleni-Maria Amaniti, Alexandra Kelman, John O. Mason, Thomas Theil

**Affiliations:** Centre for Integrative Physiology, University of Edinburgh, Hugh Robson Building, George Square, Edinburgh, EH8 9XD, United Kingdom; Université Lyon, FRANCE

## Abstract

Formation of the lateral olfactory tract (LOT) and innervation of the piriform cortex represent fundamental steps to allow the transmission of olfactory information to the cerebral cortex. Several transcription factors, including the zinc finger transcription factor Gli3, influence LOT formation by controlling the development of mitral cells from which LOT axons emanate and/or by specifying the environment through which these axons navigate. *Gli3* null and hypomorphic mutants display severe defects throughout the territory covered by the developing lateral olfactory tract, making it difficult to identify specific roles for *Gli3* in its development. Here, we used *Emx1Cre*;*Gli3* ^*fl/fl*^ conditional mutants to investigate LOT formation and colonization of the olfactory cortex in embryos in which loss of *Gli3* function is restricted to the dorsal telencephalon. These mutants form an olfactory bulb like structure which does not protrude from the telencephalic surface. Nevertheless, mitral cells are formed and their axons enter the piriform cortex though the LOT is shifted medially. Mitral axons also innervate a larger target area consistent with an enlargement of the piriform cortex and form aberrant projections into the deeper layers of the piriform cortex. No obvious differences were found in the expression patterns of key guidance cues. However, we found that an expansion of the piriform cortex temporally coincides with the arrival of LOT axons, suggesting that *Gli3* affects LOT positioning and target area innervation through controlling the development of the piriform cortex.

## Introduction

Olfaction plays a central role in the behavior of mammals with the brain receiving olfactory input from the olfactory bulb for processing. The olfactory bulb (OB) contains three principal cell types: projection neurons (mitral and tufted cells), local inhibitory interneurons (periglomerular and granule cells) and glia [1]. The mitral and tufted cells extend axons to the telencephalon, forming the lateral olfactory tract (LOT). LOT axons project over the cortical surface to innervate olfactory cortex structures including the piriform cortex [2,3]. The piriform cortex is the principal olfactory cortical area that receives monosynaptic input from the olfactory bulb through the LOT.

Correct formation of the lateral olfactory tract and specific innervation of the piriform cortex are prerequisites for the transmission of olfactory information [1,4,5]. Both of these processes are thought to depend on intrinsic properties of olfactory projection neurons that regulate axon outgrowth as well as environmental cues that control LOT axon navigation to the different structures of the olfactory cortex. This external control requires a number of axon guidance molecules, including EphrinA5 [6], Netrin1 [7] and Sema3B and F [8,9,10], as well as guidance by lot cells that are positioned on the telencephalic surface along the path followed by LOT axons [11]. In addition, LOT formation is controlled by several transcription factors [12,13,14], including the zinc finger transcription factor Gli3 which is expressed in progenitor cells of the dorsal and ventral telencephalon and in olfactory bulb progenitors but not in neurons of the piriform cortex or in mitral cells [15] (S1 Fig). *Gli3* null (*Gli3* ^*Xt/Xt*^) and *Gli3* hypomorphic (*Gli3* ^*Pdn/Pdn*^) mouse mutants both show severe defects in the formation of the olfactory system [15,16,17]. Both mutants show no discernible olfactory bulb protrusion but form an olfactory bulb like (OB-like) structure containing mitral cells and OB interneurons in ectopic dorsal or lateral positions in the telencephalon [18]. In addition, *Gli3* ^*Pdn/Pdn*^ mutants show apoptosis of precursor mitral cells in the OB-like structure [17] with residual surviving mitral cells creating a slender LOT [16,17]. Moreover, *Gli3* ^*Xt/Xt*^ mutants show severe telencephalic patterning defects resulting in the clustering of lot guidepost cells [19] and an expansion of the paleocortex [20]. Based upon these phenotypes, *Gli3* could affect LOT development by controlling intrinsic OB development, the formation of environmental cues guiding LOT axons and/or the development of the LOT target area but the severity of the defects complicate the analysis of *Gli3*’s roles in LOT formation. To circumvent these difficulties, we made use of *Emx1Cre;Gli3* ^*fl/fl*^ (*Gli3* ^*cKO*^) conditional mutants [21] which we have previously shown to have an expanded piriform cortex [22]. These mutants formed an OB-like structure that did not protrude from the telencephalic surface but contained mitral cells and olfactory interneurons. Mitral cell axons formed a LOT which occupied a medially shifted position. LOT axons innervated an extended area of the piriform cortex and their collaterals penetrated deeper layers. No obvious defects were found in the expression of telencephalic guidance cues or in the formation of lot cells consistent with the formation of the LOT. However, time course analysis confirmed that the paleocortical primordium expanded from E13.5 onwards, coinciding with the arrival of the LOT axons. These findings suggest an important role for *Gli3* in correctly positioning the LOT and controlling its innervation of the piriform cortex.

## Materials and Methods

### Mice

All mice were bred in-house in line with Home Office, UK, legislation. The licence authorising this work was approved by the University of Edinburgh’s Ethical Review Committee on 22^nd^ September 2008 (application number PL35-08) and by the Home Office on 6th November 2008. Animal husbandry was in accordance with the UK Animals (Scientific Procedures) Act 1986 regulations. *Emx1Cre* [23], *Gli3* ^*fl/fl*^ [24] and *ROSA26CAG dual stop EGFP reporter (RCE)* [25] mice were kept on a mixed background, and were interbred. *Emx1Cre;Gli3* ^*fl/+*^ mice were mated with *Gli3* ^*fl/fl*^ mice to obtain *Emx1Cre;Gli3* ^*fl/fl*^ conditional mutant embryos. Likewise, *Emx1Cre;Gli3* ^*fl/+*^ mice were mated with *Gli3* ^*fl/fl*^;*RCE* females to obtain *Emx1Cre;Gli3* ^*fl/fl*^;*RCE* conditional mutant embryos. *Emx1Cre;Gli3* ^+/+^ and *Emx1Cre;Gli3* ^*fl/+*^ embryos and animals with and without the *RCE* reporter were used as controls. Embryonic (E) day 0.5 was assumed to start at midday of the day of vaginal plug discovery. To minimise animal suffering, pregnant dams were culled by cervical dislocation under terminal anaesthesia according to the Code of Practice for Humane Killing of Animals under Schedule 1 to the Animals (Scientific Procedures) Act 1986 issued by the Home Office and embryos were removed immediately. For each marker and each stage, 3–5 embryos derived from independent litters were analysed.

### In Situ Hybridization and Immunohistochemistry

Antisense RNA probes for *Ap2ε* [26], *EphrinA5* [27], *ER81* [28], *Gad67* [29], *Netrin1* (Genepaint NM_008744), *Nrp2* [30], *Id2* [31], *Sema3F* [32], *Tbx2*.*1* [18] were labelled with digoxigenin. In situ hybridisation on 10 μm serial paraffin sections of mouse brains were performed as described [33]. Images were taken on a LeicaDMLB upright compound microscope.

Immunohistochemical analysis was performed as described previously [33] using antibodies against the following antigens: Ctip2 (1:1000, Abcam); Calretinin (CR) (1:1000, CHEMICON); lot1 (1:1000, kindly provided by Tatsumi Hirata); Map2 (1:1000; Sigma); Satb2 (1:50, Abcam); Tbr1 (1:400, Abcam); Tbr2 (1:1000, Chemicon). Primary antibodies for immunohistochemistry were detected with Alexa- or Cy2/3-conjugated fluorescent secondary antibodies. For counter staining TOPRO-3 (1:2000, Invitrogen) or Dapi (1:3000, Molecular Probes) was used. Fluorescent images were taken on a LeicaDM 5500 B fluorescent microscope. For *Gad67*/Calretinin double labelling, sections were first stained for *Gad67* mRNA followed by DAB immunohistochemistry for Calretinin as described previously [34].

### Carbocyanine Dye Placements and Analysis

DiI crystals (D282, Molecular Probes) were placed in target areas of PFA fixed, E18.5 brains using glass capillaries. Brains were incubated in the dark at 37°C in 4% PFA for 4–6 weeks. Brains were rinsed in PBS, embedded in agarose and sectioned coronally on a vibratome at 100 μm. Sections were cleared in 9:1 glycerol:PBS solution containing the nuclear counter-stain TOPRO3 (0.2 μM) overnight at 4°C. After mounting in 9:1 glycerol:PBS, sections were examined under epifluorescence microscopy with a rhodamine filter using a Leica confocal microscope.

## Results

### Mitral Cells Are Specified in *Gli3* ^*cKO*^ Mutants but Morphogenesis of the OB Is Disrupted

*Gli3* ^Xt/Xt^ null mutants [20] show an extension of the paleocortex coupled with severe patterning defects in the telencephalon leading to the formation of an highly abnormal olfactory bulb like structure which is misplaced in the dorsorostral telencephalon [18]. These severe defects in OB development as well as in the LOT target area significantly complicate the analysis of *Gli3*’s role in LOT formation. To further address these roles, we made use of *Emx1Cre;Gli3* ^*fl/fl*^ conditional mutants (*Gli3* ^*cKO*^ mutants) which we have recently shown to have an expanded piriform cortex without the severe patterning defects observed in *Gli3* ^Xt/Xt^ mutants [22].

**Fig 1:**
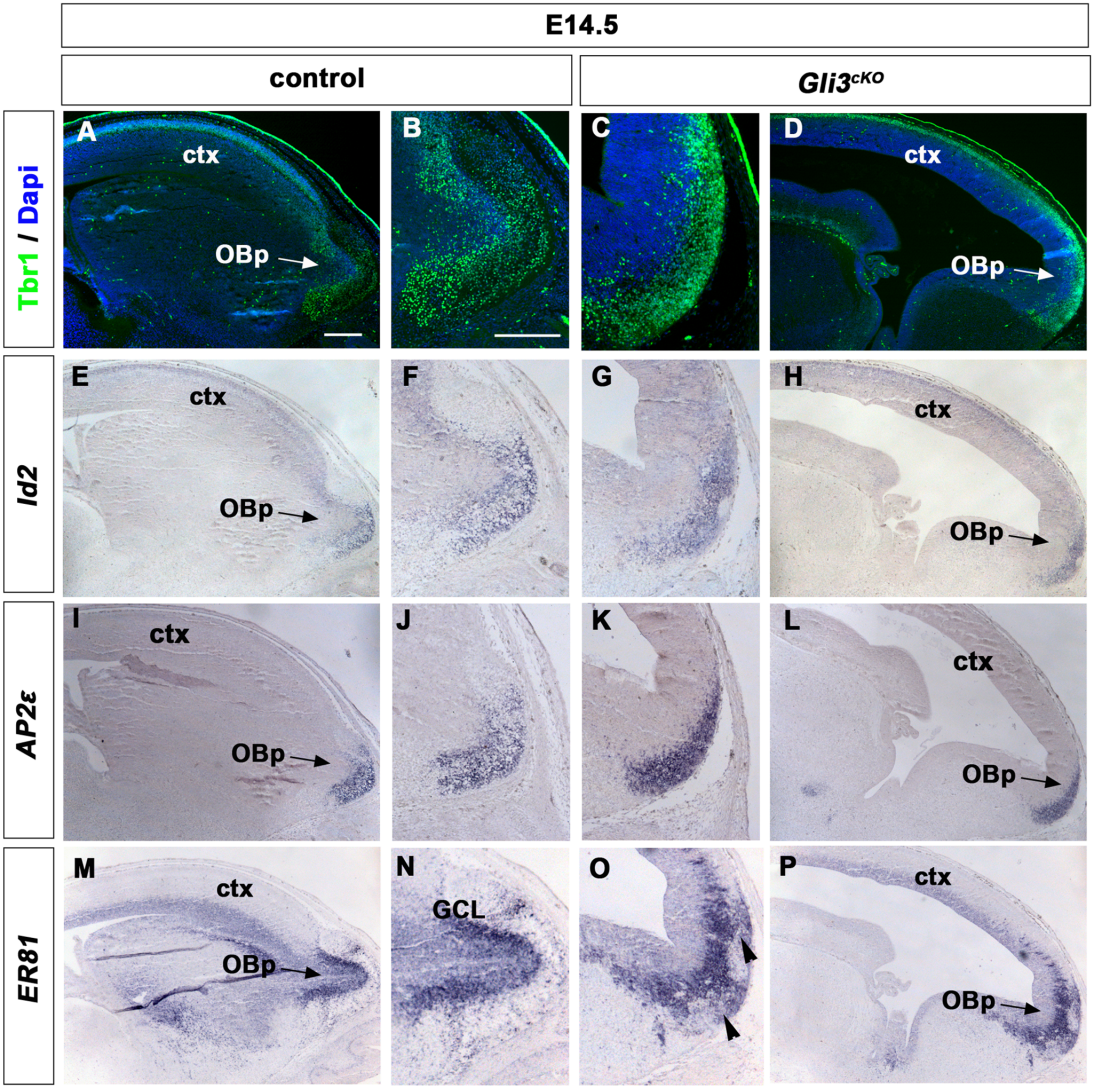
Mitral and granule cell layers are formed in the early OB-like primordium of E14.5 *Gli3* ^*cKO*^ embryos. **(A, B, E, F, I, J)** In the olfactory bulb primordium (OBp), Tbr1, *Id2* and *Ap2e* are specifically expressed in mitral cells at the rostral tip of the OB of control embryos. **(C, D, G, H, K, L)** In *Gli3* ^*cKO*^ mutants, Tbr1, *Id2* and *Ap2e* expressing cells form a thick band at the rostral tip of the OB-like primordium. **(M, N)** Control embryos show *ER81* expression in interneuron progenitors in the granule cell layer of the OB primordium. **(O-P)** In *Gli3* ^*cKO*^ mutants, *ER81* transcripts are present in a distinct cell layer of the OB-like primordium but the *ER81*+ inner cell layer is extended into the outer mitral cell layer (arrowheads in O). Scale bars: A-P:250μm.

Previous findings in *Gli3* mutants reveal the formation of an OB-like structure in abnormal positions [17,18]. Therefore, we first tested whether OB specification occurred in *Gli3* ^*cKO*^ brains. In E14.5 control brains, expression of Tbr1 [34], activating enhancer binding protein-2e *AP2e* [26] and inhibitor of DNA binding-2 (*Id2*) [26,31,34] marked mitral cells located in the outer layer of the OB primordium which clearly protrudes from the telencephalic surface (Fig 1A, 1B, 1E, 1F, 1I and 1J). In *Gli3* ^*cKO*^ brains, Tbr1, *AP2e* and *Id2* expression identified a thick band of cells at the rostral end of the telencephalon, however, no morphological protrusion was discernible (Fig 1C, 1D, 1G, 1H, 1K and 1L). We further tested specification of OB cell types by examining the formation of the inner OB layers consisting of interneurons [1]. In control brains, the ETS transcription factor gene *ER81* [35,36] is expressed in interneuron progenitors in the granule cell layer of the OB and in cortical progenitors at the ventricular surface (Fig 1M and 1N). In *Gli3* ^*cKO*^ mutants, *ER81* transcripts were present in cortex and in interneuron progenitors which form a discernible cell layer in the rostral most telencephalon. However, some *ER81* expressing cells formed ectopic clusters in the outer mitral cell layer (Fig 1O and 1P). These findings suggest that *Gli3* ^*cKO*^ mutants lack a morphological protrusion of the OBs but form mitral cells and interneurons which are organized in an OB-like primordium in a region of the rostral telencephalon resembling its normal position.

**Fig 2:**
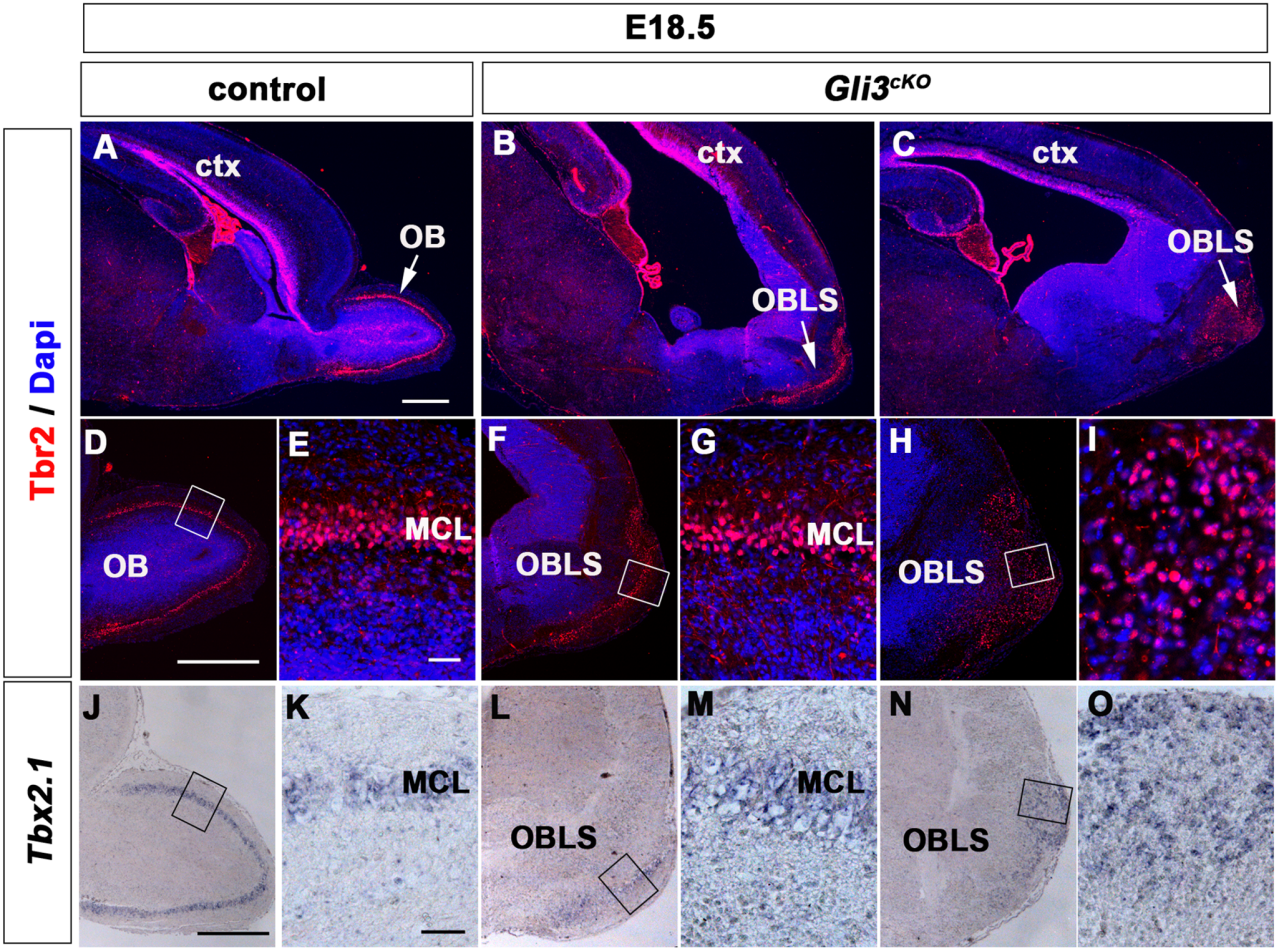
Mitral cells are present in an OB-like structure in E18.5 *Gli3* ^*cKO*^ mutant brains. **(A, D, E)** In control brains, Tbr2+ cells are present in the cortical ventricular zone and in the mitral and granule cell layers (arrowhead) of the OB which protrudes from the anterior end of the telencephalon. **(B, C, F-I)** In *Gli3* ^*cKO*^ brains, Tbr2+ cells form a discernible mitral cell layer (B, F, G) or a cluster in an OB-like structure which does not form a protrusion (C, H, I). **(J, K)** *Tbx2*.*1* expression is restricted to mitral cells in control brains. **(L-O)** In *Gli3* ^*cKO*^ brains, *Tbx2*.*1* expressing mitral cells are present in the OB-like structure and either form a layer (L, M) or a cluster (N, O). Abbreviations: MCL, mitral cell layer; OB, olfactory bulb; OBLS, olfactory bulb like structure. Scale bars:A-D, F and H:250μm; E, G and I:50μm; J, L and N:100μm; K, M and O:50μm.

To investigate later stages of OB development, we performed immunofluorescence analysis for T-box transcription factor Tbr2 in both E18.5 control and *Gli3* ^*cKO*^ brains. In control brains, Tbr2 was expressed at lower levels in granular layer interneurons of the OB and in basal progenitors of the cortex (Fig 2A) and strongly expressed in differentiated OB projection neurons with mitral cells forming a distinct layer (Fig 2D and 2E). While *Gli3* ^*cKO*^ brains still lacked a morphological visible protrusion of the olfactory bulbs, Tbr2+ cells were clearly identifiable in an OB-like structure at the rostral telencephalic end where they either formed a discernible layer (n = 2/3) (Fig 2B, 2F and 2G) or a large cluster (n = 1/3) (Fig 2C, 2H and 2I). To confirm the presence of mitral projection neurons, we further examined the expression of *Tbx2*.*1*, a T-box transcription factor gene, specifically expressed in mitral cells [37] (Fig 2J and 2K). In *Gli3* ^*cKO*^ mutant brains, *Tbx*2.1 was expressed at either a discernible mitral cell layer or a cluster of cells (Fig 2L and 2O). Thus, mitral cells that will project axons through the LOT to the telencephalon are formed in an OB-like structure, yet some *Gli3* ^*cKO*^ mutants display OB lamination defects with mitral cells forming a large cluster.

### The Lateral Olfactory Tract Innervates the Expanded Piriform Cortex in *Gli3* ^*cKO*^ Mutants

**Fig 3:**
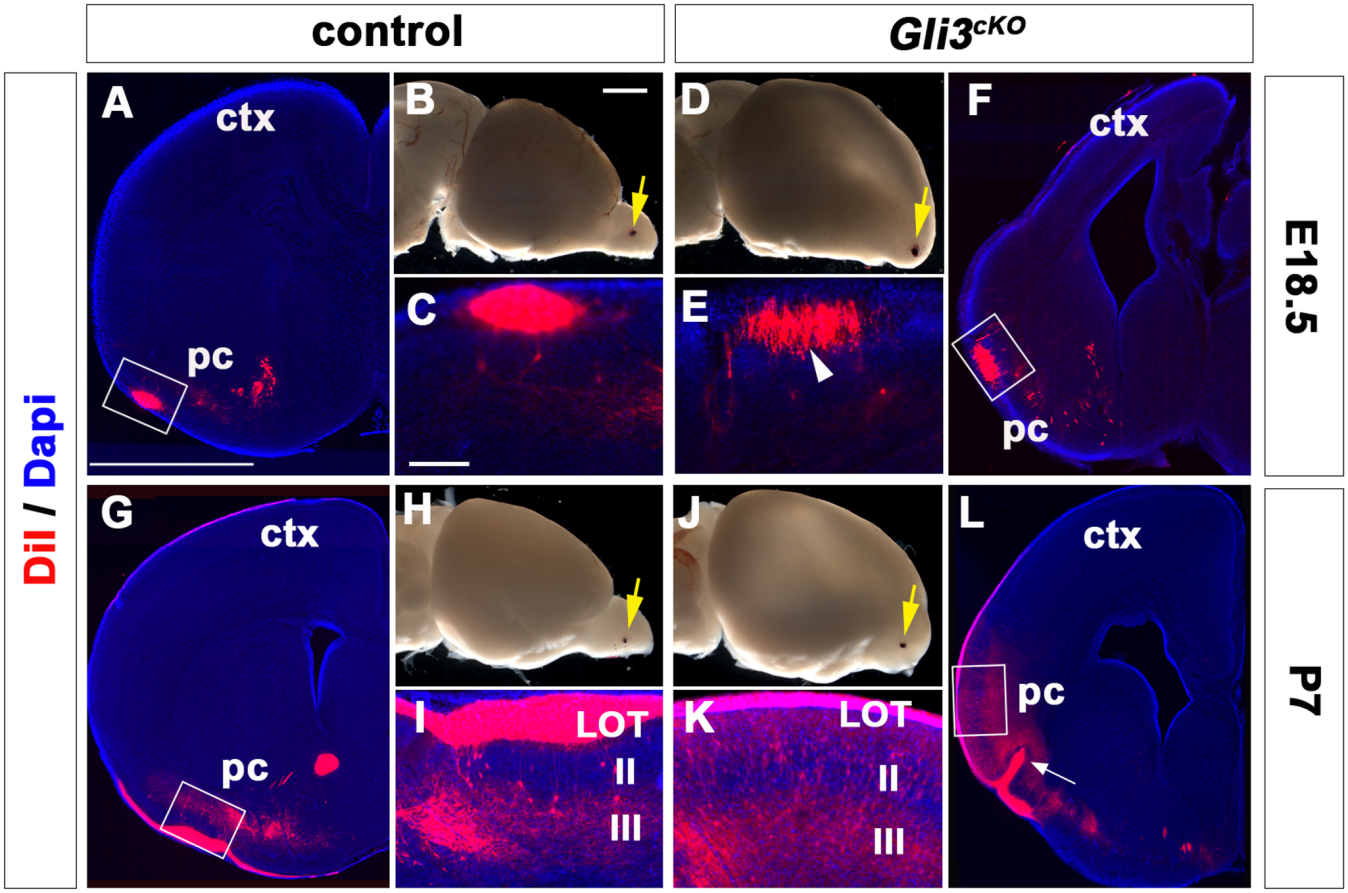
Afferent input from the olfactory bulb expands into more medial positions in the *Gli3* ^*cKO*^ cortex. **(B, D, H, J)** DiI crystal placement in the olfactory bulb (OB) and OB-like structure of control (B, D) and *Gli3* ^*cKO*^ brains (H, J), respectively (arrow). (J) In P7 *Gli3* ^*cKO*^ brains, the OB protrusion is more prominent compared to E18.5 but not as much as in wild-type brains. **(A, C, G, I)** Anterograde labeling of LOT axons and their collateral branches in E18.5 (A, C) and P7 (G, I) control brains. In P7 brains (G, I), the LOT occupies the outer piriform cortex layers and DiI labelling extends into layer III. Note the distinct gap between DiI labelling in the LOT and in layer III (I). **(E, F)** In E18.5 *Gli3* ^*cKO*^ brains, the LOT position is shifted medially and the LOT formation appears lense densely packed (arrowhead). **(K)** A dense population of DiI labelled branches is present in layer III with a barely discernible gap between the LOT and layer III (K, arrowheads). **(L)** Mitral cell axons occupy a medially expanded region in P7 *Gli3* ^*cKO*^ brains. Note the aberrant formation of an axon bundle that projects into the ventral telencephalon (L, **arrow**). Scale bars: A, G, F and L:50μm; B, D, H and J:0.5μm;C-E, I-K:250μm.

After having identified mitral projection neurons and their localization in the OB-like structure we next analyzed their axonal projection patterns through the LOT towards the piriform cortex. In mice, mitral cell axons reach the telencephalon at around E13.5 and LOT collaterals begin to colonize the piriform cortex three days later at around E16.5 [3,38]. We examined LOT formation in *Gli3* ^*cKO*^ mutant brains by placing a crystal of the lipophilic tracer DiI in the olfactory bulb and in the OB-like structure of E18.5 control and *Gli3* ^*cKO*^ mutant brains, respectively. DiI crystal placement in the OB of control brains reveals the position of the LOT in the ventro-lateral side of the telencephalon and the collateral branches that are sent off by mitral cell axons to innervate the olfactory cortex (Fig 3A–3C). In *Gli3* ^*cKO*^ brains, the LOT was also formed and the axons extended collateral branches. However, the LOT appeared less densely packed and its position is shifted medially in the mutants (Fig 3E and 3F).

The functional connectivity of the piriform cortex continues to develop during postnatal stages. In contrast to *Gli3* ^Xt/Xt^ and *Gli3* ^*Pdn/Pdn*^ embryos, *Gli3* ^*cKO*^ mutants survive postnatally allowing us to examine LOT formation at P7 using DiI tracer to label mitral cell axons anterogradely. In P7 control brains, the LOT site occupies a more extended region than at E18.5 with dense DiI labelling in layer III of the piriform cortex (Fig 3G and 3I) [39]. In P7 *Gli3* ^*cKO*^ brains, the OB-like structure is more prominent than at prenatal stages, yet an OB protrusion is not fully formed (Fig 3J). Although the LOT is formed, the DiI labelling in the piriform cortex appears more disorganized with only a barely discernible gap between the LOT and layer III in *Gli3* ^*cKO*^ brains (Fig 3K). Interestingly, mitral cell axons occupy a medially extended region (Fig 3L) coinciding with the previously described piriform cortex expansion [22]. Moreover, an aberrant axon bundle projects into the ventral telencephalon (Fig 3L, arrow). Collectively, these experiments established that mitral cell axons extended through the LOT to the telencephalon and projected collaterals into the piriform cortex of *Gli3* ^*cKO*^ mutants but the LOT was shifted medially, the innervation area was extended and aberrant DiI labelling in the deeper layers of the piriform cortex was observed.

### Laminar Organization of the Expanded Piriform Cortex Is Not Affected in *Gli3* ^*cKO*^ Mutants

**Fig 4:**
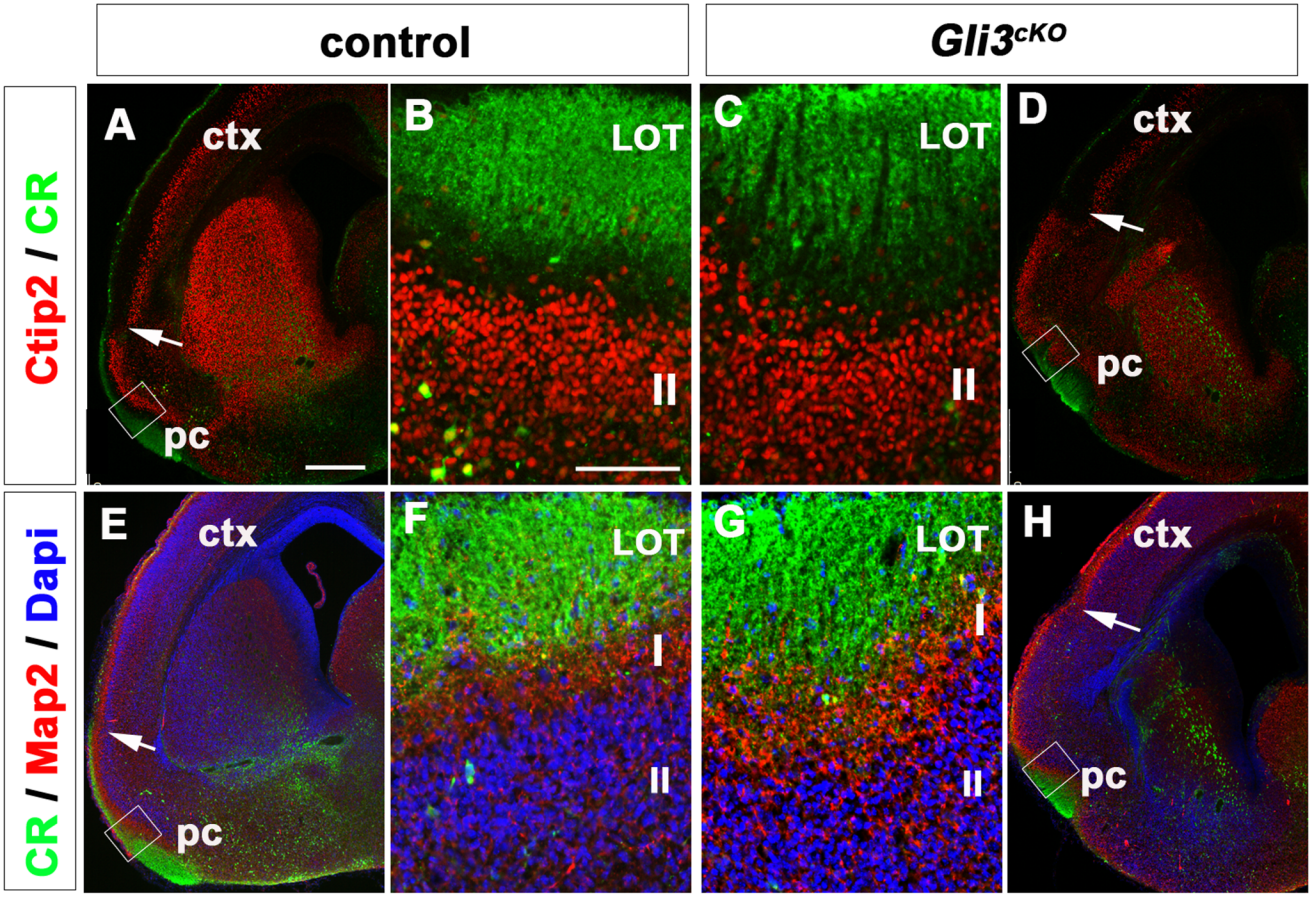
The expanded piriform cortex showed no obvious lamination defects in E18.5 *Gli3* ^*cKO*^ mutants. **(A)** Ctip2 labels neocortical neurons in neocortical layer V and layer II neurons in the piriform cortex in control brains. CR labels mitral cell axons comprising the LOT. **(B)** Higher magnification revealing the position of Ctip2+ layer II neurons with respect to the CR+ axons of the LOT. **(C-D)** In *Gli3* ^*cKO*^ mutants, Ctip2+ cells occupy layer II in the piriform cortex below the CR+ axons. **(E, F)** Organization of the piriform cortex visualized by immunofluorescence analysis for CR, Map2 and Dapi. CR labels the LOT axons; Map2 identifies dendrites in layer I and the dense population of cells in layer II is stained with Dapi. **(G, H)** In *Gli3* ^*cKO*^ mutants, no obvious defects are observed in piriform cortex organization. Abbreviations: ctx, neocortex; LOT, lateral olfactory tract; pc, piriform cortex. Arrows in A, D, E and H indicate the rhinal fissure and the transition from neocortex to piriform cortex. Scale bars: A-H:250μm.

To further investigate the relationship between the expansion of the piriform cortex and the medial shift of the LOT, we performed immunofluorescence analyses for Ctip2 and Calretinin (CR) at E18.5. Ctip2 labels layer V neurons in the neocortex and layer II neurons in the piriform cortex [40] while CR labels mitral cell axons [34] (Fig 4A and 4B). In control brains, the transition of Ctip2+ staining from layer V to layer II revealed the position of the rhinal fissure [41] (Fig 4A, arrow). Ctip2+ cells were positioned in layer II of the piriform cortex and the CR+ LOT was located in the ventro-lateral telencephalon, ventral to the transition between neocortex and piriform cortex (Fig 4A and 4B). In *Gli3* ^*cKO*^ mutants, the Ctip2+ layer II of the piriform cortex was expanded medially as indicated by the position of the rhinal fissure and the medial shift of the CR+ LOT axons (Fig 4C and 4D). Combined analysis for CR, microtubule associated protein-2 (Map2) and nuclear counterstain (Dapi) enabled further delineation of the piriform cortex layers with respect to mitral axon innervation. CR identified mitral cell axons, Map2 stained dendrites that span across layer I and dense Dapi+ cell bodies occupied layer II [42] in both control and *Gli3* ^*cKO*^ mutants (Fig 4E and 4H). Overall, these data indicate that a medial shift of the LOT correlates with an expansion of the piriform cortex whose lamination shows no apparent malformations in E18.5 *Gli3* ^*cKO*^ mutant brains.

**Fig 5:**
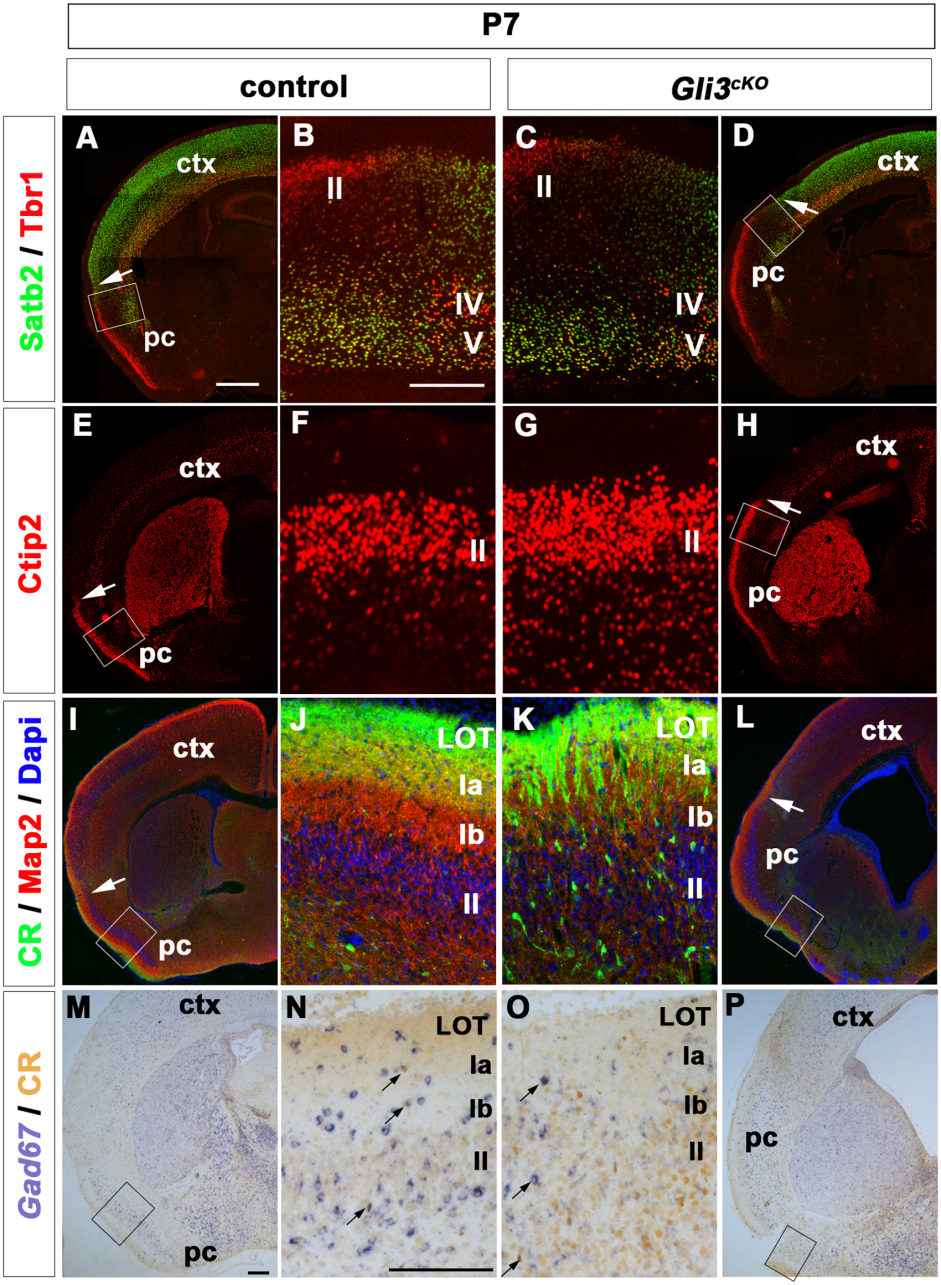
Innervation of the piriform cortex is disorganized in P7 *Gli3* ^*cKO*^ brains. **(A)** In P7 control brains, Satb2 is expressed in layer II/III neocortical neurons and Tbr1 in layer VI neocortical neurons and layer II neurons of the piriform cortex. **(B)** Magnification of the transition area from neocortex to piriform cortex. **(C, D)** In *Gli3* ^*cKO*^ brains, the transition between neocortex and piriform cortex is shifted medially. **(E, F)** Ctip2+ neurons are positioned in layer II of the control piriform cortex. **(G, H)** In *Gli3* ^*cKO*^ brains, Ctip2+ neurons occupy a similar layer position but layer II is expanded medially. **(I, J)** Cellular organization of the piriform cortex visualized by immunofluorescence analysis of CR, Map2 and Dapi. In control brains, CR+ LOT axons extend to layer Ia; Map2 dendrites are present in layer Ia and Ib and Dapi+ cells occupy layer II. **(K, L)** In *Gli3* ^*cKO*^ brains, CR+ LOT axons extend in layer Ia but some CR+ axons aberrantly project in layer Ib and II. Map2+ dendrites are disorganized in layers Ia and Ib. (**M**, **N**) *Gad67* in situ hybridization and Calretinin immunolabeling revealed interneurons and LOT axons in the piriform cortex of control brains, respectively. (**O**, **P**) In *Gli3* ^*cKO*^ brains, there is no overlap in Gad67 and Calretinin staining in layer II of the piriform cortex. Arrows in A, D, E, H, I and L indicate the position of the rhinal fissure, arrows in N and O indicate *Gad67*+CR+ interneurons. Scale bars: A-P:250μm.

The functional connectivity between the piriform cortex, the olfactory bulbs and other paleocortical structures is shaped during early postnatal stages. As the aberrant projections of mitral axons within the piriform cortex as revealed by our DiI labelling could be caused by lamina defects within the piriform cortex, we also investigated its laminar organization at P7 by immunofluorescence analysis for Satb2, Tbr1 and Ctip2. In P7 control brains, Satb2+ neurons were present in layers II/III of the neocortex but absent from the piriform cortex and Tbr1 stained layer VI neocortical neurons and layer II neurons of the piriform cortex and the olfactory tubercle [43] (Fig 5A and 5B). In addition, Ctip2 labeled layer V neocortical neurons and layer II neurons of the piriform cortex (Fig 5E and 5F). The rhinal fissure and hence the boundary between neocortex and piriform cortex can therefore be determined by the end of the Satb2 expression domain and by the change from Tbr1 and Ctip2 expression in neocortical layers VI and V, respectively, to their layer II expression in the piriform cortex. In *Gli3* ^*cKO*^ mutant brains, the layered expression of Satb2, Tbr1 and Ctip2 in the neocortex and piriform cortex was maintained. This analysis also confirmed the previously identified medial shift in the position of the rhinal fissure (Fig 5C, 5D, 5G and 5H) [22].

Based on the fact that synaptic networks between the piriform cortex and other paleocortical structures are being established at P7, we examined CR, Map2 and Dapi expression. In P7 control brains, CR+ mitral cell axons extended further from the LOT to layer Ia, but not further into layer Ib [44], to synapse with dendrites and pyramidal cells [45]. The dendritic marker Map2 labeled layer Ia and Ib and Dapi marked the dense population of cells in layer II [42] (Fig 5I and 5J). In *Gli3* ^*cKO*^ mutant brains, CR+ LOT axons were disorganized and extended beyond layer Ia to layer Ib and some even to layer II (Fig 5K and 5L). Moreover, Map2+ dendrites were more disorganized in layer Ia and in layer Ib (Fig 5K). Layer II cells were present without obvious defects although a few CR+ axons projected aberrantly into layer II (Fig 5K and 5L) as confirmed by double staining for Calretinin and *Gad67* which allowed for distinction between CR+ axons and CR+ interneurons (Fig 5M–5P). Taken together with the findings from the DiI labelling experiments, these data indicate that laminar organization of neuronal cell bodies in the piriform cortex shows no obvious defects in E18.5 or P7 *Gli3* ^*cKO*^ mutant brains. However, in both prenatal and postnatal brains, the LOT innervates an expanded piriform cortex with LOT axons projecting aberrantly into layers Ib and II of postnatal brains.

### Normal Localization of Guidance Cues in E12.5 *Gli3* ^*cKO*^ Mutant Telencephalon

**Fig 6:**
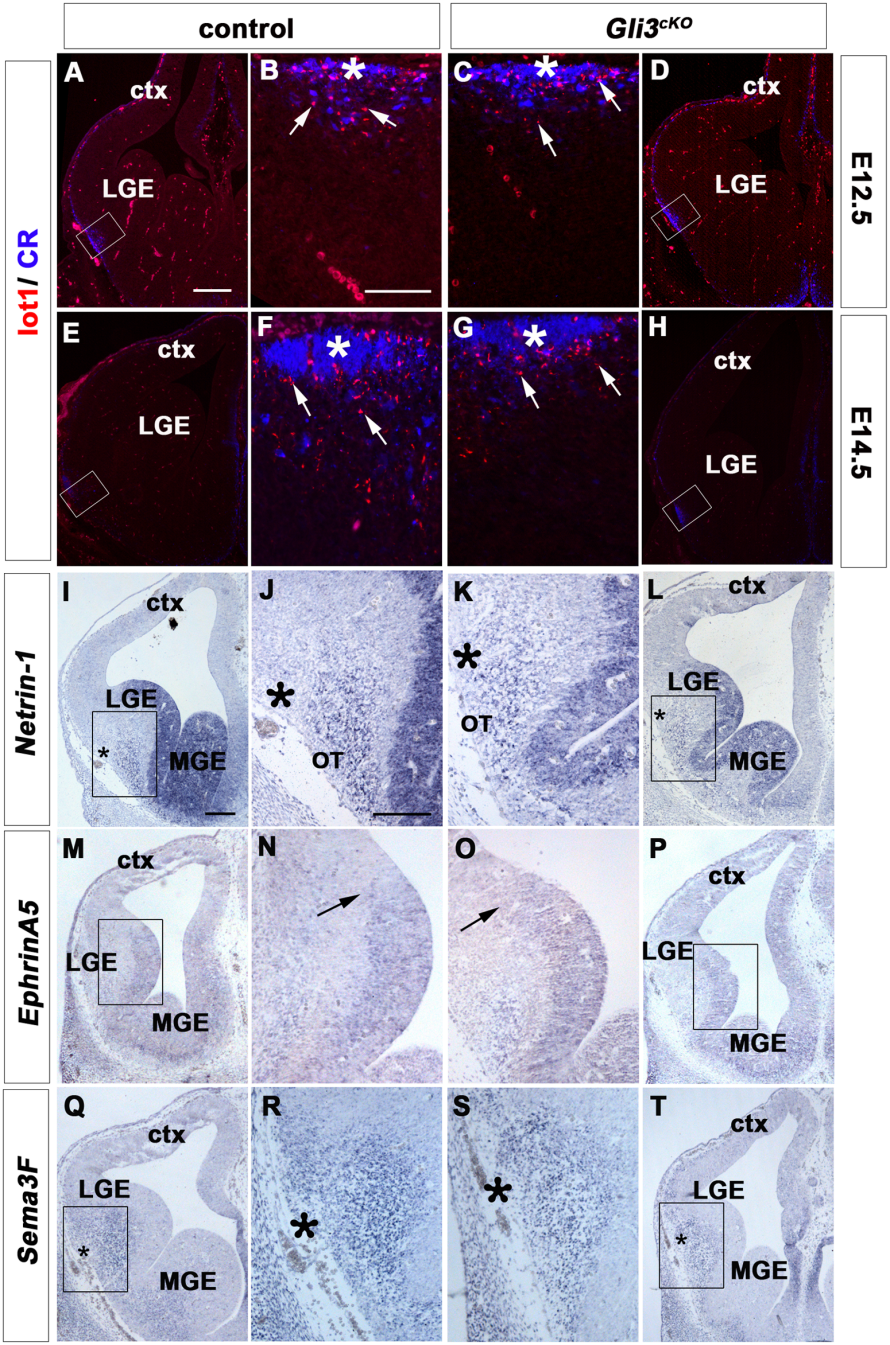
Lot cells flank the LOT axons in *Gli3* ^*cKO*^ mutant brains. **(A-H)** Immunofluorescence analysis for lot1 and CR to label lot cells and LOT axons, respectively, in E12.5 and E14.5 control and *Gli3* ^*cKO*^ mutant brains. **(A, B, E, F)** In control brains, lot1+ cells have migrated to the future LOT site at E12.5 (arrows in B) and flank the LOT site at E14.5. At this stage, lot1+ cells are also present below the LOT entry site (arrows). **(C, D, G, H)** In E12.5 and E14.5 *Gli3* ^*cKO*^ mutants, lot1+ cells flank the prospective LOT site with no obvious defects (arrows in C and G). **(I, J, M, N)** *Netrin*-*1* and *EphrinA5* transcripts are mainly identified in the neuroepithelium of the ganglionic eminences of control brains. *Netrin*-*1* is also expressed at the olfactory tubercle. **(K, L, O, P)** *Gli3* ^*cKO*^ mutants show no obvious defects in *Netrin*-1 and *EphrinA5* expression. **(Q-T)** *Sema3F* expression is confined to the LGE mantle layer but absent from the prospective LOT in both E12.5 control and *Gli3* ^*cKO*^ mutants. Abbreviations: LGE, lateral ganglionic eminence; MGE, medial ganglionic eminence. Scale bars: A-H: 250μm; I-T: 100μm. Asterisks (*) indicate the prospective LOT position in E12.5 brains and the LOT site in E14.5 brains.

Next, we started to investigate the molecular and cellular mechanisms underlying the medial shift of the LOT. The overall positioning of the LOT is controlled by a unique population of cells called “lot cells” that restrict the trajectory of the developing LOT axons [11] and by secreted signalling molecules e.g. *Sema3F* that provide repulsive cues [8,9,10]. As lot cells are widely distributed over the entire dorsal telencephalon in small clusters in *Gli3* ^*Xt/Xt*^ mutants [19] lot cell migration defects may provide an explanation for the medial shift of the LOT axons in *Gli3* ^*cKO*^ mutants. Lot cells are first detectable at the prospective LOT site in E12.5 embryos by immunofluorescence analysis using a lot1 antibody [11]. In both control and *Gli3* ^*cKO*^ brains, lot cells flank the prospective LOT site at the ventro-lateral aspect of the telencephalon with no obvious defects in the mutants (Fig 6A–6D). At E14.5 when the mitral cell axons have arrived at the LOT site, lot1+ cells were present surrounding the LOT axons with some lot1+ cells located in deeper layers of control brains (Fig 6E and 6F). In *Gli3* ^*cKO*^ brains, lot1+ cells were also detected flanking the LOT axons and ventral to the LOT (Fig 6G and 6H). Overall, lot cells were positioned in the correct location surrounding the LOT axons.

Lot cell migration is controlled by several guidance cues including *Netrin*-*1*, which guides lot cells to surround the LOT site [7] and *EphrinA5*, which prevents lot cells migrating into the subpallium [6]. In *situ* hybridization analysis for *Netrin*-*1* and *EphrinA5* in E12.5 control and mutant brains revealed *Netrin*-1 and *EphrinA5* expression restricted to the neuroepithelium of the ganglionic eminences and *Netrin*-1 expression extending to the surface of the olfactory tubercle (Fig 6I, 6J, 6M and 6N). Finally, *Sema3F* which is expressed at deep levels of the subpallium surrounding the LOT region provides repulsive cues to the LOT axons preventing them from invading the cortical plate and the ganglionic eminences (Fig 6Q and 6R) [8]. In *Gli3* ^*cKO*^ mutants, no obvious defects were detected in these expression domains (Fig 6K, 6L, 6O, 6P, 6S and 6T). Collectively these data indicate that the expression of the guidance cues that control the migration of lot1 cells and of LOT axons early in development show no obvious defects in *Gli3* ^*cKO*^ embryos.

### Expansion of the Paleocortex Precedes Entry of Mitral Cell Axons to Their Target Region

**Fig 7:**
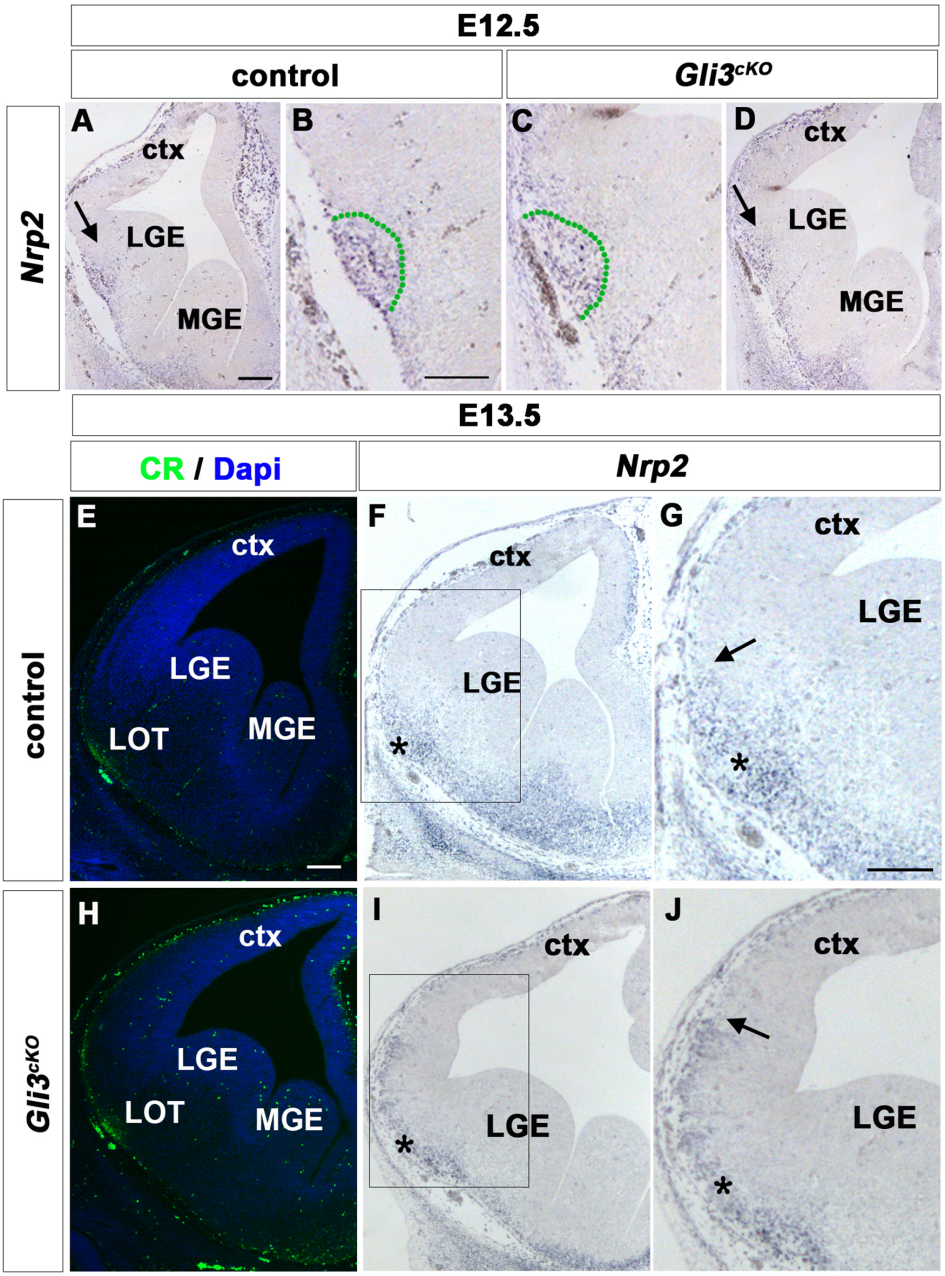
The piriform cortex is expanded at the arrival of LOT axons in *Gli3* ^*cKO*^ mutants. **(A-D)** At E12.5, *Nrp2* is expressed in the presumptive paleocortex (green dotted region in B and D) and in lot cells of both control (A, B) and *Gli3* ^*cKO*^ brains (C, D). **(E, H)** Calretinin labels mitral cell axons and indicates the position of the LOT at the ventro-lateral margin of the telencephalon in E13.5 control (E) and *Gli3* ^*cKO*^ mutant embryos (H). **(F, G)** In control embryos, *Nrp2* expression is confined to neurons of the piriform cortex and to lot cells while the area dorsal to the LOT (*) is *Nrp2* negative. **(I, J)** *Nrp2* expression expands dorsally beyond the LOT site in E13.5 *Gli3* ^*cKO*^ mutants. The arrows in (G and J) indicate the dorsal boundary of the *Nrp2* expression domain. Scale bars: A-J:100μm.

Cues produced by the piriform cortex are suggested to control its colonization by LOT axons [46]. Hence, a medial expansion of the piriform cortex before or coinciding with the arrival of LOT axons could result in a medial shift of the LOT. We therefore investigated the temporal correlation between LOT formation and the expansion of the piriform cortex in *Gli3* ^*cKO*^ mutants. Previously, we had analysed its size and position in E14.5 *Gli3* ^*cKO*^ embryos [22], i.e. one day after the arrival of LOT axons at the telencephalon. We therefore investigated piriform cortex formation earlier in development by examining *Nrp2* expression [47]. In E12.5 control brains, *Nrp2* was expressed in the presumptive paleocortex and in lot cells [9] and *Gli3* ^*cKO*^ mutants showed no apparent defects in *Nrp2* expression (Fig 7A–7D). Next, we analysed embryos at E13.5 when LOT axons arrive at the telencephalon. In control embryos, the LOT position at the ventro-lateral aspect of the telencephalon was identified by immunofluorescence analysis for CR labelling LOT axons (Fig 7E). In *Gli3* ^*cKO*^ mutants, CR+ axons occupied an identical position (Fig 7H). In contrast, *Nrp2* expression showed a remarkable difference. In control embryos, *Nrp2* expression was identified in lot neurons and neurons of the piriform cortex located at or ventral to the LOT, respectively, whereas the region dorsal to the LOT was devoid of *Nrp2* transcripts (Fig 7F and 7G). However, *Nrp2* expression was detected dorsal to the LOT in *Gli3* ^*cKO*^ embryos (Fig 7I and 7J). Taken together with our finding that lot1+ cells were still confined to the LOT site in E14.5 *Gli3* ^*cKO*^ mutants (Fig 6), these data indicate that in E13.5 *Gli3* ^*cKO*^ mutant brains the presumptive piriform cortex is already expanded when the mitral cell axons arrive at the LOT site.

## Discussion

Formation of the LOT and correct colonisation of the piriform cortex by LOT collaterals is crucial for the transfer of olfactory information. Here, we analyzed the development of this tract using *Gli3* ^*cKO*^ mouse mutants. We show that these mutants form an OB-like structure which does not protrude from the surface of the telencephalon. Despite this malformation, mitral cells are specified and produce axons that correctly reach the piriform cortex, however, the LOT becomes medially shifted. LOT collaterals colonise the expanded piriform cortex though they innervate deeper layers. While no obvious defects were found in the expression of LOT guidance cues, our time course analysis confirmed an expansion of the paleocortical primordium from E13.5 onwards, coinciding with the arrival of the LOT axons. Hence, *Gli3* is likely to control LOT positioning and target innervation by regulating piriform cortex development.

### Branching of LOT Axons Is Disorganized in *Gli3* ^*cKO*^ Mutants

Previous analyses of *Gli3* ^*Xt/Xt*^ and *Gli3* ^*Pdn/Pdn*^ mouse mutants revealed defects in formation of the olfactory bulbs, in positioning of the lot cells and in the development of the LOT target area, the piriform cortex [11,15,16,17,18]. Therefore, *Gli3* could control LOT formation by cell intrinsic mechanisms, by regulating the environment through which LOT axons migrate or by a combination of both but the complexity of the phenotypes precluded further evaluating the underlying mechanisms. *Gli3* ^*cKO*^ mutants generally show milder axon pathfinding defects in comparison to other *Gli3* mutants [21,22,48,49] and their analysis allowed a better understanding of the *Gli3* controlled mechanisms underlying LOT development. Mitral cells project axons through the LOT pathway to the telencephalon as early as E12.5 and these axons reach the LOT site at E13.5 [38,50]. LOT axons project collateral branches that innervate the olfactory cortex from E15.5 onwards and the piriform cortex first receives olfactory bulb input at E16.5 [3]. Here, we show that in E18.5 *Gli3* ^*cKO*^ brains the LOT is present but its position is shifted medially. DiI labelling in combination with Calretinin immunostainings revealed that the mitral cell axons innervate a larger area in the P7 cortex in the mutant coinciding with the enlargement of the piriform cortex. Moreover, these axons abnormally branch/extend into the inner piriform cortex layers that are normally populated by dendrites. Thus, in *Gli3* ^*cKO*^ mutants the LOT acquires an altered position and LOT axons have colonization defects.

### Mitral Cells Are Specified in *Gli3* ^*cKO*^ Mutants

Various transcription factors have been implicated in mitral cell specification and axon outgrowth. For example, in *Tbr1* null mutants the mitral cells are missing, as is the LOT [34] and in *Lhx2* null mutants mitral cells are specified but no LOT axons project to the telencephalon [14]. Here, our marker analysis provides evidence that mitral cells are specified in *Gli3* ^*cKO*^ brains despite the lack of a normal OB. In fact, the OB-like primordium occupies a region that resembles its normal position but does not protrude. This finding is in contrast to *Gli3* ^*Xt/Xt*^ and *Gli3* ^*Pdn/Pdn*^ mouse mutants where the OB-like structure is ectopically formed in more dorsal and lateral regions [18]. Moreover, E18.5 *Gli3* ^*cKO*^ mutant brains have two distinct phenotypes with some embryos forming a mitral cell layer while others form a cluster of mitral cells at the OB-like structure. The later phenotype possibly is the result of the defective OB-like primordium that was observed earlier in development. However, the LOT abnormalities were observed with 100% penetrance irrespective of the mitral cell phenotype. Moreover, displaced OBs have also been reported in *Pax6* mutants in which LOT projections nevertheless form [13]. Thus, a lack of the OB protuberance and the clustering of mitral cell do not by themselves explain LOT malformation.

### Guidance Molecules and Guidepost Cells in *Gli3* ^*cKO*^ Mutants

Several telencephalic guidance cues have been implicated in the formation of the LOT and the intracortical connections of the olfactory cortex. Regarding LOT formation these cues involve the lot guidepost cells [11] and various guidance molecules with repulsive activity such as *Sema3F* or with attractant activity such as *Sema3B* [8]. Here, we provide evidence that in *Gli3* ^*cKO*^ mutant brains, formation of the lot cells and their migration to the prospective LOT site occurs as normal. Moreover, expression of *Sema3F* and that of the *Netrin-1* and *EphrinA5* guidance cues that determine the final position of lot cells [6,7] show no obvious defects. In fact, lot cells occupy their normal position as early as E12.5 before the arrival of the LOT axons and do not form clusters in *Gli3* ^*cKO*^ mutant brains as found in *Gli3* ^*Xt/Xt*^ mutants [19]. Therefore, abnormal development of LOT guidance cues is unlikely to underlie the medial shift of the LOT.

### Altered Development of the Piriform Cortex Plays a Major Role in the LOT Axon Pathfinding Defects of *Gli3* ^*cKO*^ Mutants

In E13.5 *Gli3* ^*cKO*^ brains, mitral cell axons project normally to the telencephalon where LOT axons encounter an already medially extended paleocortex. This time course suggests that the medial shift of the LOT is secondary to the enlargement of the piriform cortex. Furthermore, correct positioning of the lot cells is not sufficient to define the position of the LOT entry site which appears to be subsequently regulated by an expansion of the piriform cortex in *Gli3* ^*cKO*^ embryos. Interestingly, the DiI labelling and Calretinin immunostainings indicate that LOT axons later innervate the whole expanded piriform cortex, yet their connections with neurons within the piriform cortex are disorganized aberrantly contacting layer II neurons. While we cannot rule out defects in the development of mitral cells, LOT axons are likely to follow guidance cues from the expanded piriform cortex. The molecular cues that trigger the formation of the LOT axon collaterals and their extension toward the olfactory cortex target are largely unknown. Guidance cues expressed at the piriform cortex have been reported to enhance axonal branching. For example Anosmin-1 has chemoattractant activity towards mitral cell axons *in vitro* and axon branches *in vivo* [46] and further experiments will be needed to clarify its importance in *Gli3* ^*cKO*^ mutants. Another interesting candidate is the membrane spanning Semaphorin Sema5B. Strikingly, the expanded piriform cortex expresses *Sema5B* in *Gli3* ^*cKO*^ mutant brains from E13.5 [22], when LOT axons arrive at the piriform cortex. *Sema5B* has recently been shown to act as a repellent towards corticofugal axons but not towards thalamic axons [51]. Moreover, *Sema5B* in combination with *Sema5A* has been reported to have a key role in establishing synaptic connectivity during postnatal retinal development with loss of *Sema5A/5B* causing the aberrant innervation of retinal layers by neurites [52]. These activities support the idea that the expanded expression of *Sema5B* in *Gli3* ^*cKO*^ mutant brains could lead to the disorganised LOT axon branching.

## Supporting Information

S1 Fig *Gli3* expression in the developing telencephalon. (PDF)
