# Supplementary material for: Cerebral Cortex Expression of Gli3 Is Required for Normal Development of the Lateral Olfactory Tract
Source: PLoS One. 2015 Oct 28;10(10):e0141525. doi: 10.1371/journal.pone.0141525 (PMC4624982; doi:10.1371/journal.pone.0141525)
Supplement: S1 Fig — (PDF) [file pone.0141525.s001.pdf]

Supplementary Figure

Supplementary Figure 1

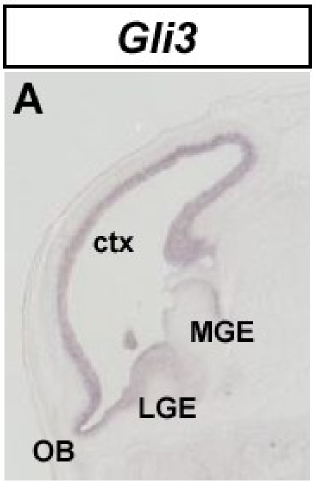

Supplementary Figure 1: *Gli3* expression in the developing telencephalon. In situ hybridization on a sagittal section through the head of an E14.5 mouse embryo. *Gli3* expression is confined to the proliferative zones of the cortex (ctx), of the medial and lateral ganglionic eminences (MGE and LGE), and of the olfactory bulbs (OB). Image was taken from Genepaint (http://www.genepaint.org).
